# Sub-10 nm Nanoparticle Detection Using Multi-Technique-Based Micro-Raman Spectroscopy

**DOI:** 10.3390/polym15244644

**Published:** 2023-12-08

**Authors:** Allan Bereczki, Jessica Dipold, Anderson Z. Freitas, Niklaus U. Wetter

**Affiliations:** Nuclear and Energy Research Institute—IPEN-CNEN, São Paulo 05508-000, Brazil; bereczki@alumni.usp.br (A.B.); jessica.dipold@alumni.usp.br (J.D.); freitas.az@ipen.br (A.Z.F.)

**Keywords:** nanoplastics, microplastics, Raman spectroscopy, emergent pollutants, titanium oxide

## Abstract

Microplastic pollution is a growing public concern as these particles are ubiquitous in various environments and can fragment into smaller nanoplastics. Another environmental concern arises from widely used engineered nanoparticles. Despite the increasing abundance of these nano-sized pollutants and the possibility of interactions with organisms at the sub cellular level, with many risks still being unknown, there are only a few publications on this topic due to the lack of reliable techniques for nanoparticle characterization. We propose a multi-technique approach for the characterization of nanoparticles down to the 10 nm level using standard micro-Raman spectroscopy combined with standard atomic force microscopy. We successfully obtained single-particle spectra from 25 nm sized polystyrene and 9 nm sized TiO_2_ nanoparticles with corresponding mass limits of detection of 8.6 ag (attogram) and 1.6 ag, respectively, thus demonstrating the possibility of achieving an unambiguous Raman signal from a single, small nanoparticle with a resolution comparable to more complex and time-consuming technologies such as Tip-Enhanced Raman Spectroscopy and Photo-Induced Force Microscopy.

## 1. Introduction

Pollution by microplastics (MPLs) is a growing global concern as vast amounts of plastics have been found in diverse environments in recent years. The oceans, acting as the primary sink for microplastics, play a crucial role in their accumulation and transport within marine ecosystems. When exposed to ambient stressors, MPLs degrade into even smaller particles known as nanoplastics [1,2,3,4,5]. Consequently, the presence of MPLs has contributed to the alarming increase in the abundance of nanoplastics (NPLs) as particle size decreases. However, given the lack of appropriate techniques through which to characterize the NPLs, they have been largely neglected [6,7,8,9,10]. The substantial quantity of NPLs, relative to MPLs, raises concerns about their potential impacts on biological systems. Although several studies have been conducted to understand the effects of NPLs on animals, environments, and biological systems, there remains a notable gap in simple and suitable techniques for quantifying and characterizing these plastic nanoparticles (NPs).

Engineered NPs represent emerging contaminants at the nanoscale range, many of which are metallic oxides or metallic NPs. Titanium dioxide NPs hold particular significance as they are extensively used in the production of cosmetics, UV protective coatings, and paints. It was estimated that the production of TiO_2_ NPs will reach 2.5 million tons by 2025 [11,12]. Consequently, as NPs are released into the environment proportionally to their usage [13], concerns regarding TiO_2_ pollution have arisen since the ocean serves as the ultimate sink for these NPs [14], thus further emphasizing the need to understand the potential consequences of their presence.

While the geometry of NPLs can be measured using technologies such as transmission/scanning electron microscopy (TEM or SEM) or atomic force microscopy (AFM), the standard spectroscopic techniques utilized for chemically characterizing MPLs, FTIR, and micro-Raman spectroscopy (MRS) fail in terms of resolution to characterize NPL samples that exhibit weak signals and are invisible to the microscope that is coupled to the equipment [7,15,16,17]. The chemical composition of NPLs is usually obtained by pyrolysis-gas chromatography-mass spectrometry (Py-GC/MS) [6,7,18,19,20,21,22]. It should be noted, however, that these approaches are destructive, and their lower detection limits do not allow the characterization of individual NPL particles. Thus, the current analytical methods available are insufficient for comprehensive analyses and furthering the understanding of NPLs.

When moving to nano-sized particles, techniques that combine scanning probe microscopy (SPM) with spectroscopy, such as Atomic Force Microscope-Infrared Spectroscopy (AFM-IR), Photo-Induced Force Microscopy (PiFM), Tip-Enhanced Raman Spectroscopy (TERS), or s-SNOM (scattering scanning near-field optical microscopy), are currently the natural candidates for characterizing individual NPs. These techniques offer the potential to characterize particles at the nanoscale with resolutions as small as 10–20 nm, and they are intrinsically limited by the radius of the metallic coatings of the tips [6,23,24,25,26,27,28]. However, even though these techniques are more interesting for the characterization of NPs, they have seen limited use in the characterization of NPLs. Only one TERS study has been conducted on a PS and polyisoprene polymer blend thin film, where 150–300 nm sized PS features were characterized [28] while PiFM was being applied to the measurement of polystyrene (PS) nanoparticles as small as 20 nm [29,30]. TERS and PiFM are relatively new technologies that have few, but expensive, instruments installed worldwide, thus resulting in a limited number of publications focusing on their application to NPLs. The time-consuming method of exploring large sample regions for nanoparticles, the expensive cost of metallic-coated tips, and the vulnerability of tips to contamination are all disadvantages of these approaches.

MRS has been extensively used as the gold standard for MPL characterization [6,15,31], and it has been demonstrated that its use is acceptable for studying NPs even at the laser diffraction limit. In fact, by using optical tweezers, 40 nm latex NPs were detected in suspension [32] and, more recently, 50 nm PS were detected by Gilibert et al. [33]. However, the direct detection of single NPLs over substrates has only been achieved more recently [34]. The detection of a single 50 nm diameter NPL was claimed by mapping the 1230 cm^−1^ peak of PS along the x and y axis of the NPL; however, only a single peak (which was not sufficient for identifying PS) at 1230 cm^−1^ was detected, with a signal-to-noise ratio (*SNR*) of less than three [34].

The detection of small PS NPLs has also been explored using surface-enhanced Raman spectroscopy (SERS). For instance, PS-NPs as small as 50 nm were detected using SERS [35,36,37,38]. However, it remains challenging to ascertain whether a single particle is being detected or if the signal originates from multiple particles or clusters once they are not geometrically characterized.

Hence, any techniques used to individually characterize NPs that can be employed within the existing capabilities of laboratories worldwide are welcome. Here, we demonstrate, for the first time, the unambiguous chemical identification of NPs from 50 nm down to 9 nm at the single-particle level. Our multi-technique approach combining MRS and AFM enables NP identification using commonly available Raman equipment that is found in labs worldwide. This development provides researchers with the means to access and analyze the properties of nano pollutants like NPLs, which have been neglected due to a lack of suitable experimental techniques. The AFM, which is primarily used for the localization of the NPs, also plays a crucial role in adding the dimensional features to this technique, thus allowing for a comprehensive and total characterization of NPs.

### AFM/MRS Precision Colocalization Technique

**Figure 1:**
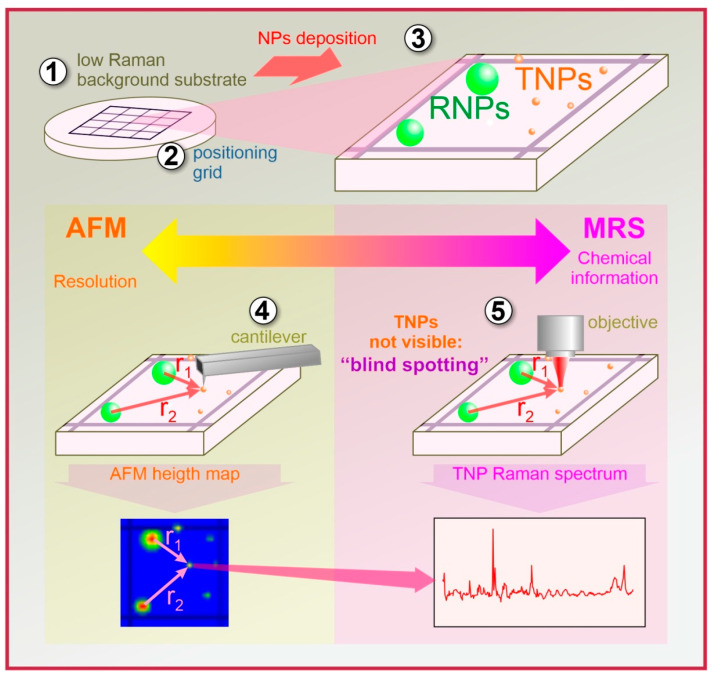
Scheme of the colocalization technique used to measure the spectra of small NPs.

The scheme of the technique developed in this work is displayed in Figure 1. This multi-technique approach, which was developed for the characterization of single nanoparticles, primarily involves a precision colocalization approach, and it is used to precisely position the nanoparticles for direct Raman spectroscopy. The technique relies on (1) a low-noise substrate (choosing a low-background substrate is crucial for distinguishing the weak signal from nanoparticles (NPs) from background noise); (2) a grid for the *coarse* colocalization of AFM and MRS; (3) the use of large reference NPs (RNPs) of an approximately 200 nm size together with small target NPs (TNPs) for *precise* colocalizations; and (4) AFM measurement followed by MRS.

Steps 2 and 3 are necessary for finding the small TNPs with the MRS, whose optical microscope is incapable of “seeing” below the diffraction limit but can still visualize the RNPs. In Step 4, when using the MRS (after AFM has been completed), we first localize the specific grid that contains the TNP in question (Step 2), then find the large RNPs, which are visible and have a strong Raman signal (Step 3). Next, using the high precision AFM coordinates and the RNPs for triangulation (as shown in Figure 1), we may “blindly” localize the TNPs with the micro-Raman laser, even though visualization via the microscope objective is no longer viable. No Raman mapping, which is unpractically time-consuming for such small particles, is needed because the Raman laser is directly positioned on top of the TNPs.

MRS resolution is fundamentally restricted by the diffraction limit, which is given by the objective’s numerical aperture (NA ≅ 1 for air objectives) and the laser wavelength, and it results in a practical NP lower size limit of approximately 250 nm. Below this size, it becomes difficult to distinguish individual NPs. Nevertheless, it is important to note that this limitation does not imply that the Raman sensor cannot detect the signal from smaller NPs [32,33,34,39,40], as modern MRS systems with their high-sensitivity EMCCD are capable of detecting weak signals even down to the single-photon level [41,42,43]. Furthermore, 200 nm sized RNPs can be measured in a relatively short time interval by MRS given their strong Raman signal, thus allowing for the fast and exact positioning of an MRS laser on top of TNPs via triangulation, which in turn allows for high integration times and therefore high-quality TNP spectra (which are ultimately limited by the drift that moves the TNPs out of the focus region).

## 2. Materials and Methods

### 2.1. Nanoparticles (NPs)

PS nanospheres (Lab261 Ltd., Palo Alto, CA, USA) with nominal diameters of 500 nm, 200 nm, 100 nm, 50 nm, and 25 nm were used as NPLs for these studies. Nominal diameters, mean diameters, the polydispersion index (PDI), and the corresponding diameter standard deviations can be found in Table S1. The NPs were supplied and dispersed in a 0.1% Tween 20 in deionized water, which contained 2 mM of NaN_3_ as an antimicrobial agent. For all NP diameters supplied, the PS concentration was 1% *w*/*v*.

Two different titanium oxide NP sizes were used, the first with a mean particle size of 410 nm (Ti-Pure^®^ R-900, Chemours, Wilmington, DE, USA), and the second with a size of 25 nm (85% rutile, 15% anatase) from Evonic (Essen, Germany) commercially named Degussa P-25 Titania.

### 2.2. Substrates

**Figure A1:**
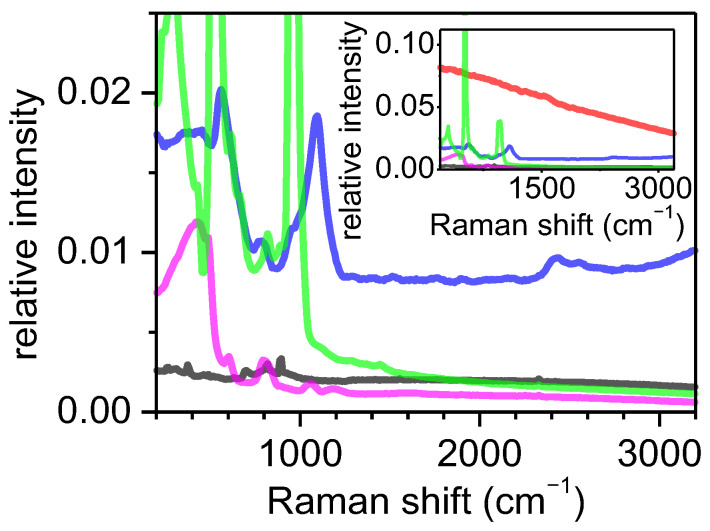
The measured Raman spectra from some of the common substrates normalized to the peak of silicon at 516 cm^−1^. Green: silicon; blue: glass slide; magenta: fused silica; and black: aluminum. Inset: increased scale to show the signal from gold (red).

Substrate plays a crucial role in the Raman detection limit when dealing with small NPs. The small spatial overlap between the NP and the laser source leads to a significant portion of the laser volume interacting with the substrate, thus generating undesired Raman scattering and fluorescence. To address this issue, a study was conducted to select a suitable substrate, as shown in Appendix A. Comparable substrate Raman intensities are presented in Figure A1. Fused silica and aluminum were found to be the overall best substrates, and while fused silica was found to be the best substrate for Raman shifts larger than 1000 cm^−1^, aluminum was found to be the best substrate for Raman shifts below 1000 cm^−1^.

Round, polished fused silica substrates of a 25 mm diameter and 3 mm thickness were used throughout our experiments. Furthermore, 500 × 500 µm^2^ grids containing 50 × 50 µm^2^ fields were either marked with the help of a femtosecond laser or a diamond tip glass cutter that was attached to a homemade translation mechanism. The field size of 50 × 50 µm^2^ was chosen because a single field could be still fully imaged with an MRS microscope (57 × 54 µm^2^ field of view with a 100×, 0.9 NA objective) and an AFM (100 × 100 µm^2^ scanning area). Images of the marked grid substrates can be found in Figure S1.

### 2.3. Solution Preparation and Deposition

For each experiment, different NP concentrations were prepared by dilution in milli-Q water, followed by vortexing. For the experiments involving Raman acquisition and allowing the individual visualization of single NPLs (Section 3.1) without the colocalization technique, a quartz substrate without a grid was mounted at the center of a spinning disk, which rotated at 300 rotations per minute. A droplet of approximately 300 nL was placed in the center of the substrate and spread to cover an area of 5–8 mm in diameter. Spinning continued for another five minutes until the complete drying of the solution was achieved.

For the experiments involving mapping or direct, blind targeting, the dilutions were adjusted to achieve an average of approximately 10–20 RNPs and 100–200 TNPs in each 50 µm × 50 µm field after deposition. Next, 300 nL of solution was dropped with a micropipette over the substrate, thus forming a droplet of about 1 mm diameter. As the droplet evaporates, many particles migrate to its edges, leading to the formation of regions with high concentrations near the droplet borders and in the regions with low concentrations in their central portion. Figure S2 shows an image of the grid after the deposition of the NPs. Solution concentrations are summarized in Table 1. Figure S3 show grids with deposited PS (Figure S3a) and TiO_2_ (Figure S3b) NPs.

### 2.4. Micro-Raman Spectroscopy (MRS)

The micro-Raman spectra were acquired using a LabRam HR system (HORIBA, Palaiseau, France). A diffraction grating with 300 grooves·mm^−1^ was used in all NPL experiments, and a 600 grooves·mm^−1^ grating was used for the TiO_2_ experiments. The laser wavelength was 532 nm. For all NPL diameters, the laser power was 6 mW because of the PS-NP degradation at higher powers, while up to 30 mW were used for the titanium oxide. A 100× and N.A = 0.9 objective was utilized for all the experiments.

The pinhole diameter was between 20 µm and 80 µm. This determination was made by gradually decreasing the pinhole diameter of the apparatus in order to reduce the background signal from the substrate, as well as by comparably increasing the NPL signal.

After each measurement, the subtraction of the substrate’s background signal was performed until its 300–500 cm^−1^ band vanished. Then, an additional baseline subtraction was performed for the final spectra.

### 2.5. Atomic Force Microscopy (AFM)

Atomic force microscopy was performed using an Omegascope SPM (HORIBA) in tapping mode with an aluminum-coated silicon tip, an 8 nm tip radius, and a 40° tip cone (HQ:NSC14/AL BS, Mikromasch, Sofia, Bulgaria).

## 3. Results and Discussion

### 3.1. Visualization and Raman Acquisition of Large NPLs

**Figure 2:**
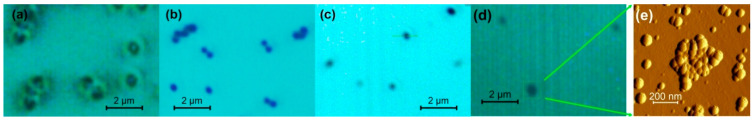
MRS optical microscope images containing single NPLs and clusters of NPLs. (**a**) 500 nm, (**b**) 200 nm; and (**c**) 100 nm. (**d**) Dark spots visible on a MRS microscope of 50 nm NPLs, and (**e**) AFM showing that the dark spots correspond to agglomerates of 50 nm NPLs.

**Figure 3:**
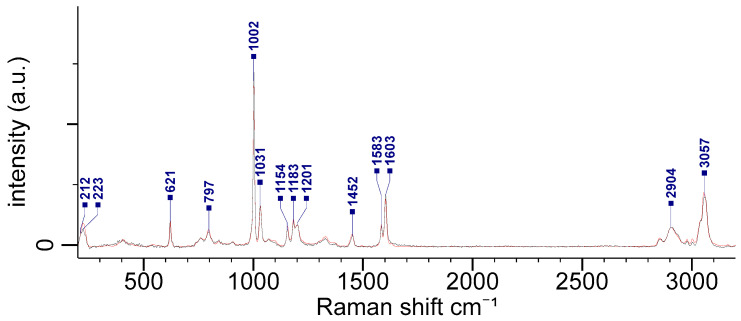
Comparison between the spectrum measured from a single 500 nm particle (black) and the reference PS spectrum (red) from the Wiley database.

The microscope visualization of large nanoplastics was previously conducted to determine the limit at which NPs can be measured without the need for the additional techniques. As shown in Figure 2, single nanoparticles of a 500 nm and 200 nm size could be observed with the MRS microscope, and 100 nm sized NPs could be seen in the microscope but not with enough resolution that they could be distinguished between single particles and clusters. Single NPs of a 50 nm size could not be visually detected. For the 500 nm sized nanoparticles, it was possible to directly target a single nanoparticle with an integration time of 500 s, thereby obtaining a high-quality spectra with an *SNR* = 257. As shown in Figure 3, a spectrum measured from a single 500 nm NPL was compared to a PS reference spectrum from the Wiley database, which was available from KnowItAll^®^ informatics system 2024 software, and a high level of agreement (92.2% correlation) between the spectra was observed. Similarly, the spectra from 200 nm and 100 nm sized NPs was obtained. However, a subsequent *SNR* analysis revealed, in some cases, that the *SNR*s for the 100 nm sized NPs were similar to the *SNR*s of the 200 nm sized NPs, thus indicating that these were in fact clusters of 100 nm NPs and not single particles. Based on these findings, we concluded that only NPs down to a 200 nm size could be visually targeted for Raman acquisition without the need for an additional AFM to ascertain their single-NP nature.

**Figure 4:**
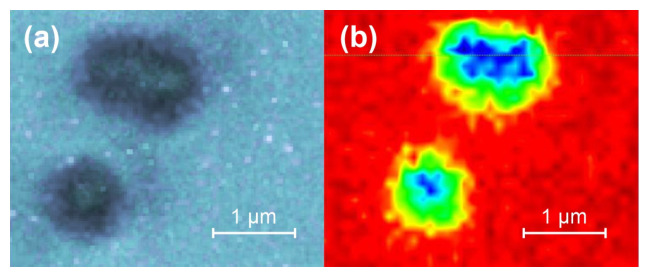
Microscope images (**a**) and the Raman intensity map (**b**) of the 1000 cm^−1^ PS peaks in 500 nm sized NPLs showing a single particle (**bottom**) and a dimer (**top**). The integration time at each point of the Raman map was 0.2 s.

A map of 500 nm sized NPLs is shown in Figure 4. Observe that the dimer (a cluster of two particles) in Figure 4a was not resolved in the Raman map of Figure 4b. Similar maps of acceptable quality for the NPLs with smaller diameters could not be achieved due to the mechanical instabilities (drift) in the system’s sample stage combined with the necessarily longer acquisition times (Appendix B).

### 3.2. Direct, Blind Targeting of NPLs

Using the MRS system microscope, it is possible (see Figure 2) to differentiate single plastic particles from clusters in the case of 500 nm and 200 nm sized particles, but for 100 nm sized NPLs, distinguishing single NPLs from clusters becomes impossible. Both the 200 nm and 100 nm sized NPLs appeared as dark dots on the microscope, which had about 250 nm sized FWHM diameters due to the blurring caused by the point spread function. The 50 nm diameter NPs initially appeared as dark spots on the microscope, but they were later found to be agglomerates of many NPLs, with the single NPs remaining invisible (Figure 2d,e). Once visualized by the MRS’s objective, the Raman spectra of NPs down to 200 nm in size were easily acquired. In a first set-up, we applied traditional Raman mapping to the 50 nm sized TNPs, but this led to unpractical long measurement times and stability-related issues (Appendix C).

In order to improve the positioning resolution, an additional xyz piezo scanner was used on top of the MRS table. Using this piezo scanner, it was possible to pinpoint the laser directly on top of the TNP without the need to scan an area of 1 µm^2^ (see Appendix C). The MRS table produced a drift of approximately 100 nm/min, even if it was turned off, which limited the acquisition time to 1 min. After this time period, the NPL would start to drift out of the MRS laser’s focal region. Details of the drift characterization can be found in Appendix B.

**Figure 5:**
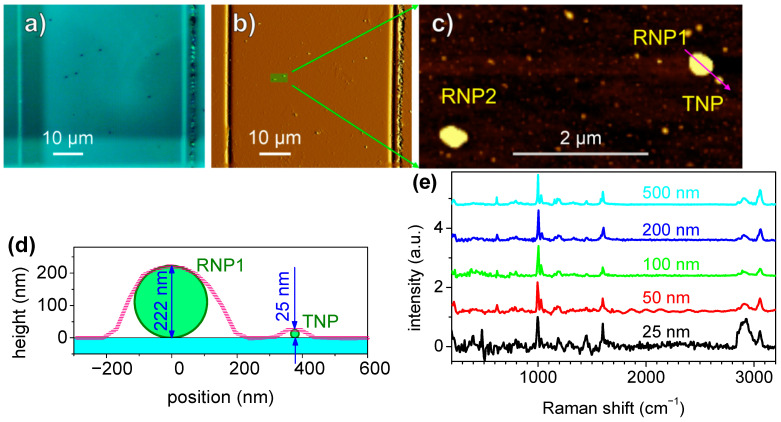
Blind targeting of 245 nm PS-NPL. Optical microscope image of a 50 × 50 µm^2^ field containing 200 nm sized RNPs and 25 nm sized TNPs (**a**) and the corresponding AFM (mag) of the same area (**b**). (**c**) The AFM zoom of the ROI highlighted in (**b**), showing two RNPs and one TNP. (**d**) The AFM height profile measured along the magenta line in (**c**), which connects the centers of the RNP1 and TNP (magenta line). The cyan-filled rectangle represents the substrate, and the green circles represent the NPs plotted in scale. (**e**) The Raman spectra measured from single NPLs, with diameters ranging from 500 nm to 25 nm.

Figure 5a,b show the optical microscope images and the AFM, respectively, of a single 50 × 50 µm^2^ field grid from which it was possible to identify the RNPs on both images. A higher-resolution AFM was performed in the highlighted region of Figure 5b, as shown in Figure 5c, where two RNPs (RNP1 and RNP2) and one TNP were identified. An AFM height profile was measured along the magenta line, as indicated in Figure 5c, and the corresponding profile is plotted in Figure 5d. From this profile, the TNP height of 25.2 ± 0.1 nm was measured. The distance between the centers of RNP1 and TNP was 376 ± 14 nm. To obtain the Raman spectrum of the TNP, the laser was first positioned on top of RNP1, and this was then translated by the displacement vector of a 374 nm length. Focusing was performed by minimizing the laser spot size that is visualized by the objective on the substrate surface at a low power (~6 µW). This positioning and focusing procedure was performed in about 10 s, followed by a 1 min spectrum acquisition. These last two steps were repeated ten times for the 25 nm TNPs to accumulate more spectra and achieve a better *SNR*. Each time, the laser was positioned again in order to keep the positioning error below 100 nm (drift). The total acquisition time was 410 s. Individual spectra can be found at Figure S4. A clear fingerprint from a single 25 nm PS-NP could be obtained with a reduction in measurement time from 6.5 h to 6.8 min when compared to colocalized Raman mapping (Appendix C).

### 3.3. Direct, Blind Targeting of TiO_2_ NPs

**Figure 6:**
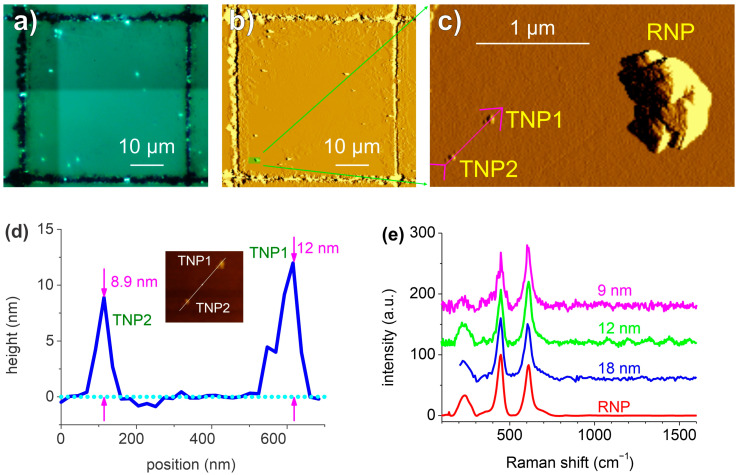
Blind targeting of TiO2 NPs. (**a**) Image of a 50 × 50 µm^2^ field that was obtained with a Raman microscope; (**b**) the corresponding AFM (mag) of the same area; (**c**) the AFM (mag) of the region highlighted in (**b**), showing a 288 nm TiO_2_ RNP and two TNPs; (**d**) the AFM height profile along the magenta line indicated in (**c**), with measured TNP sizes of 8.9 and 12 nm; and (**e**) the TiO_2_ spectra showing 445 cm^−1^ and 608 cm^−1^ peaks, which were measured from the RNP (30 s acquisition) (red) and from the TNPs (average of 3–5 spectra acquired with 1 min each) with diameters of 18 nm (blue), 12 nm (green), and 9 nm (magenta).

Next, the same colocalization procedure was applied to engineered TiO_2_ NPs. The approximately 300 nm sized titanium oxide RNPs appeared as strong, visible white dots under the MRS optical microscope, as seen in Figure 6a. The AFM image of the same 50 × 50 µm^2^ field containing RNPs is shown in Figure 6b. An AFM image of the highlighted region in Figure 6b, containing a 288 nm high RNP and two TNPs is shown in Figure 6c. The AFM height profile along the line indicated in Figure 6c is shown in Figure 6d, and it contains two TiO_2_ TNPs of 12.0 ± 0.1 nm and 8.9 ± 0.1 nm size.

The final spectra, after substrate spectra and baseline subtractions, are shown in Figure 6e, where the spectrum from a RNP is used as a reference.

Being a ceramic, rutile NPs can withstand much higher laser powers (30 mW was used) than PS NPLs. Furthermore, the Raman cross-section of rutile is much higher than PS, thus resulting in an even more intense Raman signal. Although the peaks of the TiO_2_ coincided with the peaks of the fused silica substrate (see Figure S5), integration times as small as 1 s were enough for the appearance of a 608 cm^−1^ peak. It was expected that even smaller particles can be detected by employing more suitable substrates that minimize substrate overlap (see Appendix A). The raw spectra are shown in Figure S5.

### 3.4. Signal to Noise Analysis

In order to determine the limit of detection (LOD) and estimate the acquisition times, a signal-to-noise (*SNR*) analysis was conducted based on the Raman spectra acquired from NPs with different diameters. For PS, the *SNR* was determined by measuring the signal height of the peaks (the measured peaks were the 1000 cm^−1^ PS aromatic ring peak and C-H stretch peaks, which were present even at the smallest measured NPLs), while the noise level was measured by measuring the RMS noise in the range of 1800 cm^−1^ to 2800 cm^−1^ (which exhibited no discernible peaks). Increasing the laser power did not enhance the *SNR* due to the degradation of the polystyrene nanoparticles at higher power levels. For TiO_2_, the peaks at 220 cm^−1^, 445 cm^−1^, and 610 cm^−1^ were measured, and the noise was measured in the 1000 cm^−1^ to 3000 cm^−1^ range.

The following considerations were used to compare the *SNR* of the spectra acquired with different acquisition times or with different confocal pinhole sizes: (i) the number of data was proportional to the product of the acquisition time and the flux of Raman photons from the NP that were passing through the confocal pinhole; and (ii) the flux of the photons from the NP passing through the confocal pinhole was proportional to the pinhole area. Thus, the *SNR* can be written as follows:

*SNR*(*t*,*d*,*P*) = *S*(*d*)·*P*·*t*^½^ (1)

where *t* is the acquisition time, *d* is the particle diameter, and *P* the pinhole diameter. *S*(*d*) = *SNR*·*P*^−1^·*t*^−½^ is the normalized *SNR*.

**Figure 7:**
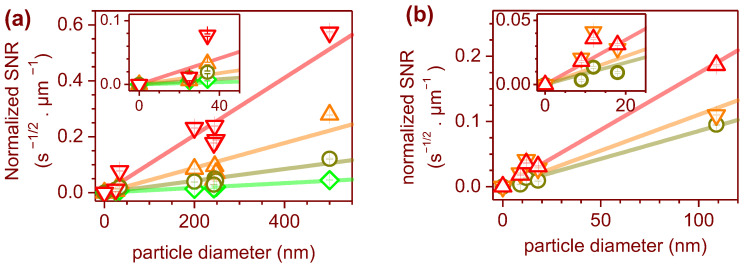
Normalized *SNR* as a function of the particle diameter, and fitted lines for the polystyrene peaks at (**a**) 1000 cm^−1^ (inverted triangles), 2850 cm^−1^ (diamonds), 2900 cm^−1^ (triangles), and 3050 cm^−1^ (circles). The TiO_2_ peaks were at (**b**) 220 cm^−1^ (circles), 445 cm^−1^ (triangles), and 610 cm^−1^ (inverted triangles). The insets show data with smaller scale.

**Figure 8:**
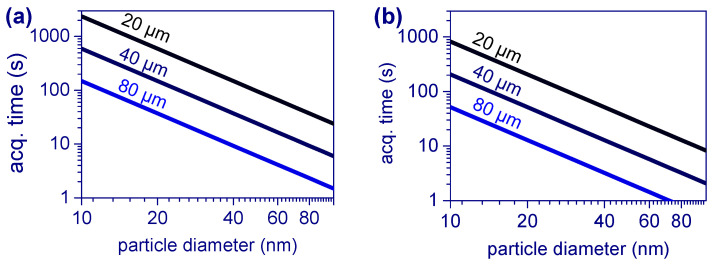
Estimated acquisition times for the 1000 cm^−1^ peak of PS, as well as for (**a**) the 445 cm^−1^ peak of TiO_2_ and (**b**) an *SNR* = 10 for pinholes ranging from 20 µm to 80 µm.

The acquisition parameters used in the experiments varied slightly with different gratings. Furthermore, they had different grating efficiencies for each Raman shift and different sensitivity configurations for the EMCCD. Nevertheless, all of the data were combined in the same plot to provide a rough estimative of the acquisition times and detection limits. To gain insight into the dependence of *S*(*d*) on the particle diameter, we plotted the normalized *SNR* as a function of the particle diameter in Figure 7. Since *SNR = S/N* must reach zero for particle diameters approaching zero, we plotted a line passing through the origin to fit the data, i.e., *S*(*d*) = *S × d*, where *S* is the slope of the fitted line. The slopes obtained were 1.03 ± 0.07 s^−1/2^ µm^−2^ for the 1000 cm^−1^ peak of PS, and 1.7 ± 0.1 s^−1/2^ µm^−2^ for the 445 cm^−1^ peak of TiO_2_. Based on these slopes, the estimated acquisition times calculated from Equation (1) were plotted in Figure 8.

**Figure 9:**
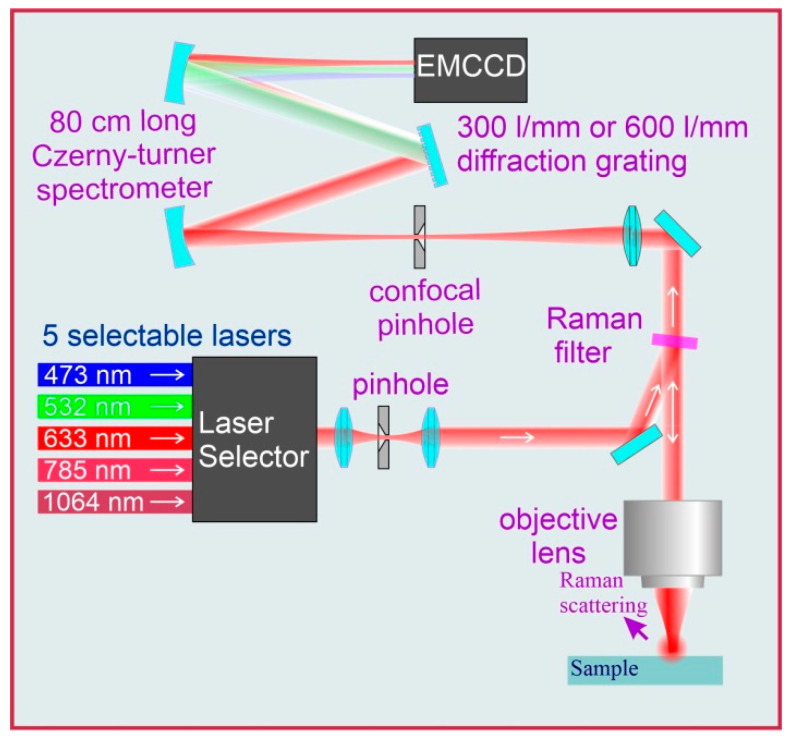
Scheme of the Raman system.

It is worth noting that the confocal pinhole has a dual function in the Raman system, serving as both a signal collection depth discriminator and a spectral resolution limiter for the spectrometer (as can be seen in the simplified schematic of the Raman system depicted in Figure 9). As the confocal pinhole size increases, more of the signal from the particle enters the spectrometer; however, the background signal from the substrate increases faster. Although a more detailed study is required to determine the optimal pinhole size, we observed an improvement in the 25 nm sized NPLs’ signals up to an approximately 80 µm pinhole diameter, even with a slight increase in the captured substrate signal. The estimated acquisition times of Figure 8 were calculated for pinhole sizes from 20 µm to 80 µm.

**Figure 10:**
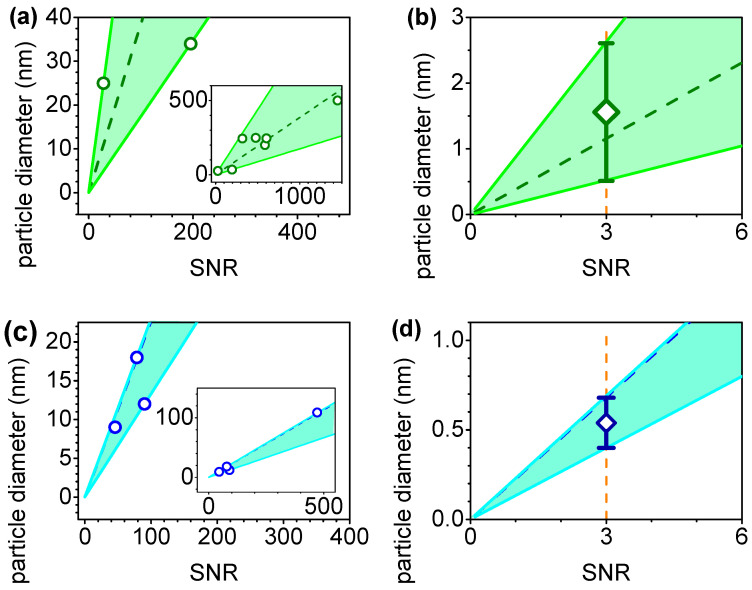
Particle diameters (circles) as a function of the *SNR* calculated for a 1000 s acquisition time that considered the 1000 cm^−1^ peak of PS (**a**,**b**) and the 445 cm^−1^ peak of TiO_2_ (**c**,**d**). The points are the experimental points that were corrected by different acquisition times and pinhole diameters. The filled regions are the sectors adjusted to encompass the highest and lowest slopes to the experimental data. The insets in (**a**,**b**) display a larger scale to show all of the data points. (**b**,**d**) are displayed with a zoomed scale. The vertical dashed line represents the limit of detection with an *SNR* = 3, and the diamonds indicate the theoretical LOD.

To estimate the achievable LOD of the technique, we considered an acquisition time of 1000 s (~16 min), which is a reasonable timeframe limited by the system’s mechanical stability and requires multiple one-minute acquisitions with repeated repositioning. The 1000 cm^−1^ peak of PS and the 445 cm^−1^ peak of TiO_2_ were considered for this purpose. By plotting the diameter as a function of *SNR* in Figure 10, we determined the LOD as the crossover of the sectors (Figure 10) at *SNR* = 3 through using Equation (1). Remarkably, the LOD for the PS and TiO_2_ were found to be 1.6 ± 1.0 nm (equivalent to 2.2 ± 1.4 zeptograms or 10^−21^ g) and 0.54 ± 0.15 nm (equivalent to 349 ± 97 yoktograms or 10^−24^ g), respectively, thus indicating the technique’s potential for detecting nanoparticles even in the sub-nanometer range. However, it should be stressed that this theoretical calculation takes into account a linear relationship between the particle diameter and the *SNR*.

**Figure 11:**
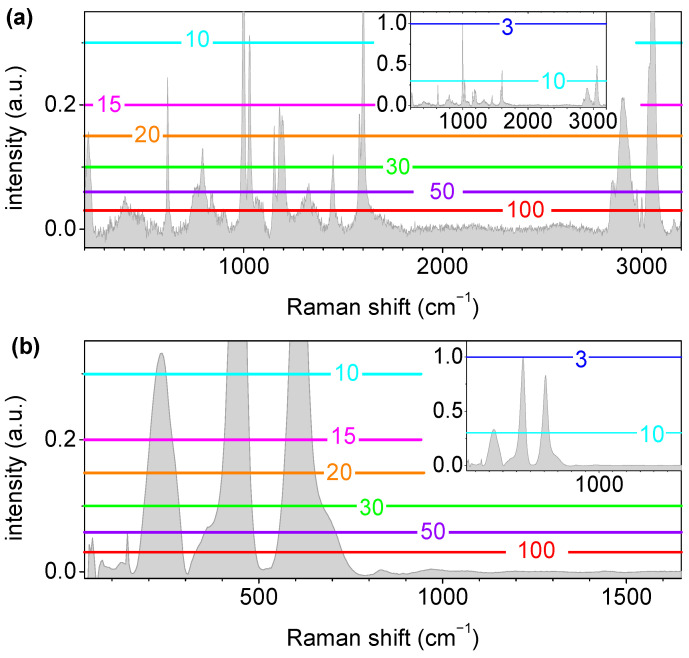
Spectra of the PS (**a**) and TiO_2_ (**b**) used to estimate which spectral lines should be visible at a given *SNR* for the most intense line (1000 cm^−1^ peak for PS and 445 cm^−1^ peak for TiO_2_). The horizontal lines, which are distinguished by different colors (cyan, magenta, orange, green, purple, and red), represent the visibility threshold, i.e., the level of *SNR* = 3, when the most intense peak’s *SNR* was 10, 15, 20, 30, 50, and 100, respectively. The inset shows the spectra at larger intensity scale.

On an additional note, it should be observed that, although *SNR* = 3 is accepted and widely used as the detection limit, a single peak might not be enough to unambiguously identify a certain type of plastic material. Therefore, higher *SNR*s might be necessary for the strongest peak to ensure the presence of several peaks, and each must have at least an *SNR* of 3. However, a *SNR* of >10 would be desirable for identifying PS, for allowing for the detection of four peaks, and for allowing for the detection of three peaks of TiO_2_, as shown in Figure 11.

## 4. Conclusions

This study demonstrates a successful technique for characterizing NPs. This was achieved by combining Atomic Force Microscopy and Micro Raman Spectroscopy via a colocalization technique that is based on the use of a positioning grid and triangulation that employs reference nanoparticles (RNPs). The technique was applied to nanoplastics (NPLs) and the engineered NPs of TiO_2_, and the unambiguous spectra of 25 nm (polystyrene NPs) and 9 nm (TiO_2_ NPs) NPs that corresponded to the mass detection limits of 8.6 attogram and 1.6 attogram, respectively, were obtained. The main limiting factor for the measurement of the smaller NPs was mechanical stability, which determined the maximum acquisition time. The resolution limit for TERS was approximately 20 nm; however, we did not encounter any work addressing the TERS measurements of NPLs of such a small size, and this was likely due to the small Raman signal emitted by these NPLs. In the case of TiO_2_, a clear spectrum for a 9 nm sized NP was achieved, thereby surpassing the TERS resolution and approaching the capability of PiFM. While this technique exhibited remarkable effectiveness in achieving these impressive detections of small NPs, it was found to be exceptionally sensitive to the alignment of the Raman system. With the slightest misalignment, the measurements of the smallest NPs could not be replicated. These challenges, coupled with the time-consuming nature of the method at this early stage, limited the quantity of data available for NP characterization. Nevertheless, this constrained dataset effectively demonstrates the potential of the technique. We aspire to overcome these challenges in future works, thus enabling the characterization of a larger number of nanoparticles.

Further enhancement of the detection limit is possible using more dedicated substrates, specifically in the case of TiO_2_, where the Raman peaks of our fused silica substrates overlapped with the TiO_2_ peaks. Among the tested substrates, aluminum demonstrated the best potential for this specific task.

Our technique offers a complete characterization, encompassing morphological, dimensional, and chemical aspects, and thereby aiding in the determination of the geometry and composition of emerging pollutants. One of the benefits of this technique is the use of standard instruments that are readily available in many laboratories worldwide. Additionally, current commercially available correlative scanning electron microscopy/MRS systems can directly utilize this technique, thus further enhancing its accessibility and applicability in nanoparticle analysis. By addressing the gap in laboratory techniques for nano pollutant characterization, this study contributes to the advancement of nanoparticle analysis. Furthermore, the technique holds promise for extending its applications beyond standardized NPs and TiO_2_ to encompass a wider range of nanoparticle samples, including the many other NPs found in the environment. The current lack of publications on NPL characterization in different environments underscores the need for techniques such as the one presented in our study. By offering the means for a better understanding and assessing the properties of NPLs, this technique can contribute to addressing the challenges posed by these emerging pollutants.

## Figures and Tables

**Table 1:** Specification of NP solutions and classification in the visual detection of single NPs, Raman mapping, and direct, blind targeting.

Detection Technique	TNP	RNP	TNP Concentration ng·mL^−1^	RNP Concentration ng·mL^−1^
Visual	PS 500 nm	None	10 × 10^6^	
Visual	PS 200 nm	None	5 × 10^6^	
Visual	PS 100 nm	None	2 × 10^6^	
Visual	PS 50 nm	None	1 × 10^6^	
Mapping	PS 50 nm	PS 200 nm	50	300
Blind	PS 25 nm	PS 200 nm	9	300
Blind	TiO_2_ 20 nm	TiO_2_ 400 nm	15	10,000

**Figure A2:**
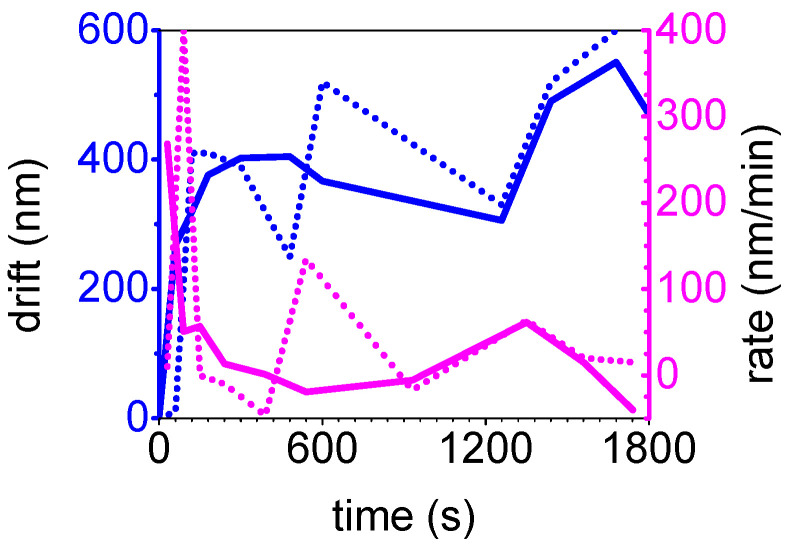
The measured drift of the piezo-driven translation stage and its corresponding drift rate. Blue: drift; magenta: rate; continuous line: xy plane; and dotted line: z axis.

**Figure A3:**
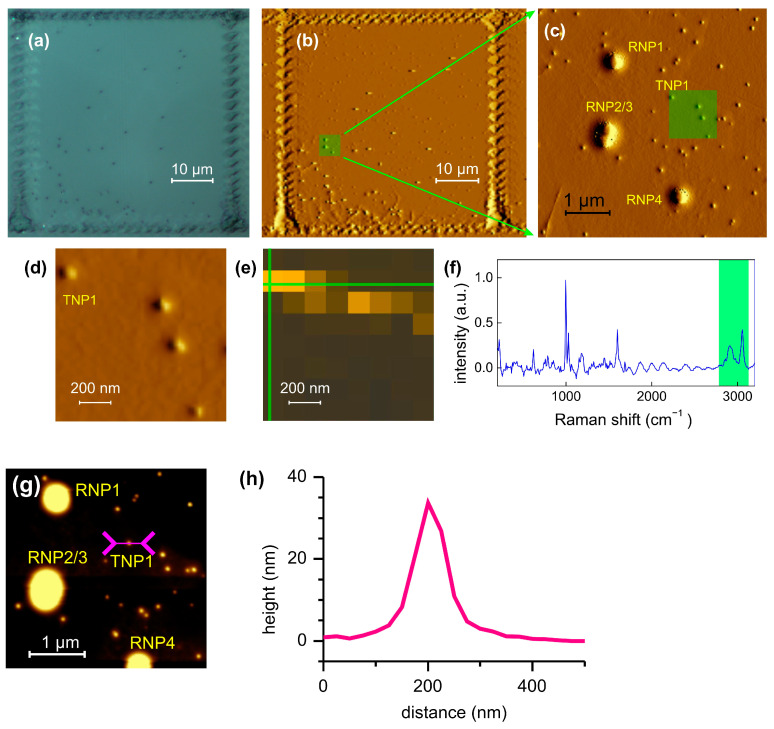
(**a**) MRS optical microscope image of a 50 × 50 µm^2^ grid field containing NPLs of a 200 nm (RNPs) and 50 nm (TNPs) size. (**b**) The AFM (magnitude) of the same field. (**c**) The AFM (mag) zoom of the region highlighted in (**b**), showing the TNPs together with the RNPs. (**d**) The AFM (mag) zoom of the highlighted region of (**c**), in which Raman mapping (**e**) was performed on the top two TNPs. (**f**) The spectrum measured at the cursor point in (**e**), and which corresponds to the spectrum of the single particle TNP1. The highlighted region in (**f**) corresponds to the spectral region selected for Raman mapping (**e**). (**g**) The AFM height measurement of the highlighted region in (**b**) with the identification of RNPs and TNP1, and (**h**) the height profile along the line identified in (**g**), which passes across the 34 nm height TNP1.

## Data Availability

The data presented in this study are available on request from the corresponding authors.

## Supplementary Materials

The following supporting information can be downloaded at: https://www.mdpi.com/article/10.3390/polym15244644/s1: Table S1: PS-NP diameters and polydispersion indexes (PDI) as informed by the manufacturer and the corresponding standard deviation for the diameters; Figure S1: Quartz substrate containing laser-marked and diamond-scratched grids; Figure S2: Images of NP depositions over the diamond-scratched grid before deposition, after deposition, and after drying; Figure S3: A 50 × 50 µm^2^ laser-marked grid after 200 nm + 25 nm PS deposition and 400 nm + 20 nm TiO_2_ NPs deposition; Figure S4: The measured 1 min spectra from the 25 nm PS-NP; Figure S5: The raw spectra obtained from TiO_2_ NPs, and the reference spectra obtained from fused silica substrates and rutile.

## Appendix A. Low Raman Background Noise Substrates for Single-Particle Detection

The substrate is critical for detecting single NPs with diameters that are smaller than the system’s diffraction limit (which arises because expressive Raman scattering and fluorescence originates from substrate areas and may be captured together with the NP signal). We performed an investigation in order to find a suitable material for use as a Raman substrate for nanoparticle detection. Some common substrates in the literature are silicon, aluminum, and gold. The Raman signals from these substrates were measured for comparison. All Raman spectra were acquired using a 532 nm laser, 60 mW, a 100×/0.9 N.A objective, a 300 µm sized pinhole, and 300 lines/mm diffraction grating, while the integration time varied from 0.6 s (fused silica) to 60 s (aluminum). The intensities of the spectra were first normalized to a 60 s acquisition time, and were then normalized to the silicon peak at 516 cm^−1^. The results are plotted in Figure A1. The gold and aluminum substrates are suitable for many applications due to the absence of peaks, i.e., they are flat after subtracting a baseline; however, the gold’s signal intensity was higher than the other substrates in most of its spectral range, which leads to higher noise levels after subtracting it from the small signals of NPs. The glass substrate peaks were in the region of interest of PS, and the Raman signal was very high, thus causing similar problems as gold. Silicon had two strong peaks at 620 cm^−1^ and 1000 cm^−1^ that interfered with PS. Fused silica was chosen over aluminum because it presented the smallest intensity above 1000 cm^−1^, which is where most of the PS Raman peaks of interest were found.

## Appendix B. Characterization of Stage Drift

The characterization of the drift was performed by tracking a 200 nm sized particle on the Raman microscope camera. The x and y positions were monitored over time, and, for each measurement, the particle’s focus was adjusted, thus enabling the measurement of the z coordinate drift. Rough focusing was achieved by moving the objective column, which was controlled by stepper motors. All subsequent z adjustments were conducted by moving the additional xyz-piezo scanner positioned on top of the microscope stage. The piezo scanner allowed for movement within a 30 µm × 30 µm range in the xy-plane, and a 6 µm range in the z-axis. The measured drifts in the xy plane (i.e., the distance from initial position) and along the z-axis together with their corresponding rates are presented in Figure A2. The drift reached 400 nm·min^−1^ in the first few minutes, and then reached up to 60 nm · min^−1^ during the rest of the duration. A 100 nm drift resulted in a 30% reduction in the Raman signal from a small NP (NPs with a diameter *d* < < 2*w*, where *w* is the 1/e^2^ laser waist). To ensure reliable measurements, a maximum acquisition time of 1 min was set to guarantee that the displacement remained smaller than 100 nm, and this was performed as the measuring occurs some minutes after the coarse stage adjustment.

## Appendix C. Colocalized Raman Map for the PS NPs of Intermediate Sizes

Raman mapping by colocalization was also tested, not for the direct targeting of a NP, but to cover a 1 µm^2^ region that contained several TNPs. By restricting the mapping region to such a small area, the completion of the map in a reasonable time became feasible despite the long integration times required. The use of colocalization was crucial because these NPs were invisible to the microscope, as well as to also ensure that the measured particles corresponded to single NPs.

In Figure A3a, a picture taken with the MRS microscope of one of the 50 × 50 µm^2^ fields of the colocalization grid containing NPS is shown. There, it was possible to see the 200 nm sized RNPs as dark spots, whereas the (nominally) 50 nm sized TNPs were not visible. By comparing Figure A3a with the AFM image of Figure A3b, one can identify the same RNPs on both images. A higher resolution AFM of a small area allowed us to identify the TNPs and RNPs (Figure A3c). A small region of interest (ROI) of 1 × 1 µm^2^ around the TNPs was Raman-mapped (scanned) with 8 × 8 acquisition points (i.e., the highlighted region of Figure A3c). Even at a lower resolution of 140 nm × 140 nm, the integration time was 1 min with six accumulations per point, thus resulting in a total measuring time of 6.5 h. Figure A3d shows a zoom of the AFM region containing TNP1, and Figure A3e shows the Raman map of the same region. Mechanical stability issues (drift) prevented a mapping of the whole area, thus resulting in a lack of signal in the lower half of the mapping area (Figure A3e). The Raman spectrum obtained from TNP1 is shown in Figure A3f, and its height profile is shown in Figure A3h, thus proving that the size of the NP was 34 nm.

Although the Raman measurement of a single TNP was achieved by mapping, the time consumed for such a tiny, scanned area and the stability problems associated with it were impracticable, especially when considering that mapping areas that are not in an AFM-colocalized set-up would have to be much larger than 1 × 1 µm^2^.
