# Supplementary material for: Sub-10 nm Nanoparticle Detection Using Multi-Technique-Based Micro-Raman Spectroscopy
Source: Polymers (Basel). 2023 Dec 8;15(24):4644. doi: 10.3390/polym15244644 (PMC10747801; doi:10.3390/polym15244644)
Supplement: Supplementary file 1 [file polymers-15-04644-s001.zip › polymers-2724194-supplementary.pdf]

Sub-10 nm Nanoparticle Detection Using Multi-Technique based micro-Raman Spectroscopy

Supplementary material

Table S1. PS-NPs diameters and polydispersion indexes (PDI) as informed by the manufacturer and corresponding standard deviation for the diameters.

nominal diameter (nm)	mean diameter (nm)	PDI	SD (nm)
500	582	0,053	134
200	248	0,021	36
100	98	0,022	14
50	50	0,063	12
25	28,9	0,109	9,5

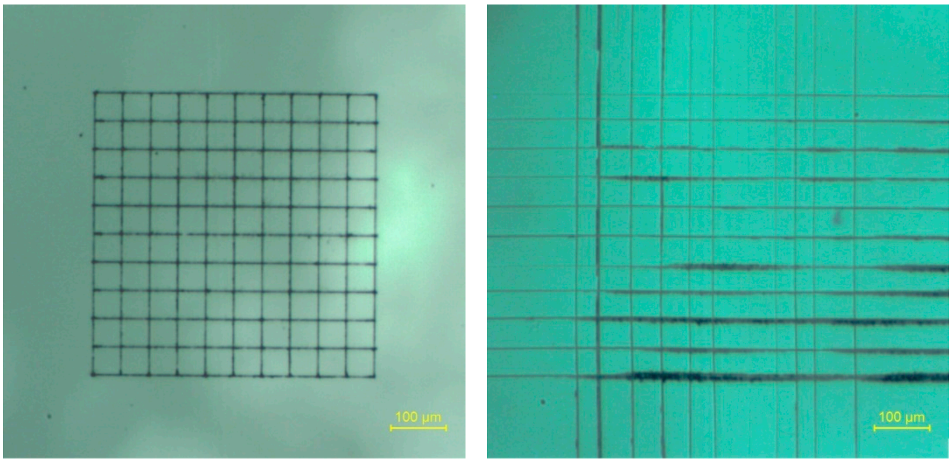

Figure S1. Quartz substrate containing laser marked (a) and diamond scratched (b) grids.

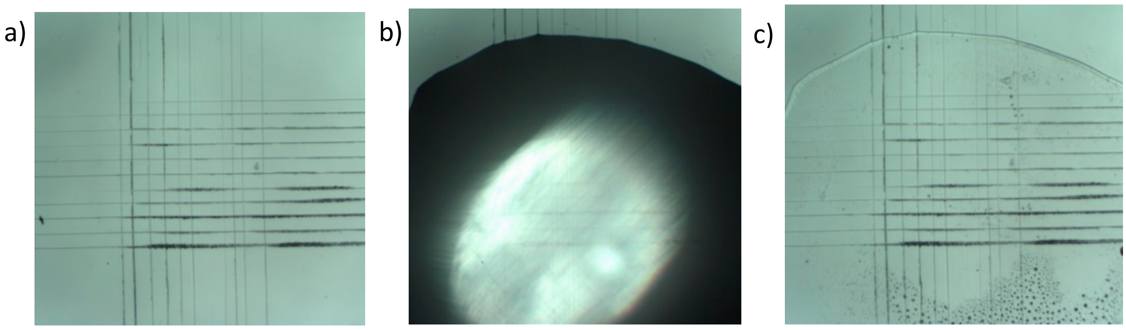

Figure S2. Images of NPs deposition over the diamond scratched grid. (a) before deposition, (b) after deposition and(c) after dried.

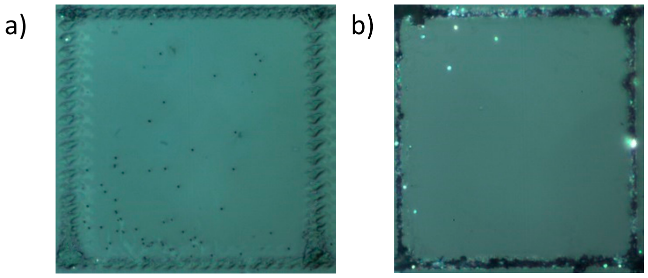

Figure S3. 50 x50 µm² laser marked grid after (a) 200 nm + 25 nm PS deposition and (b) 400 nm + 20 nm TiO_2 NPs deposition.

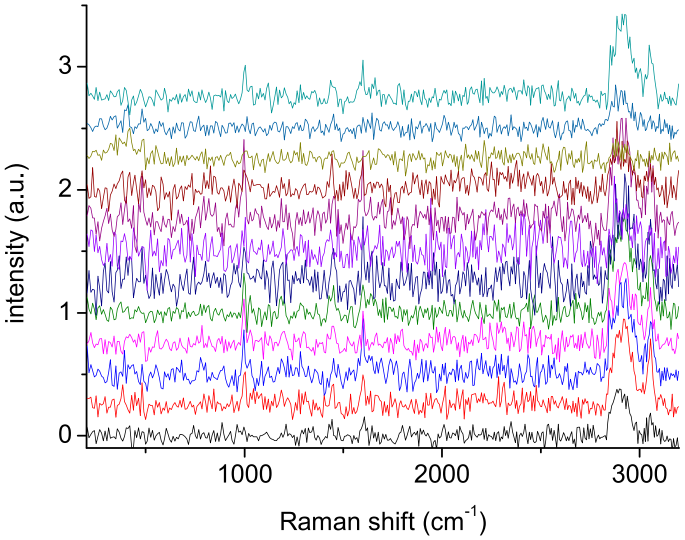

Figure S4. Measured 1 min spectra from the 25 nm PS-NP.

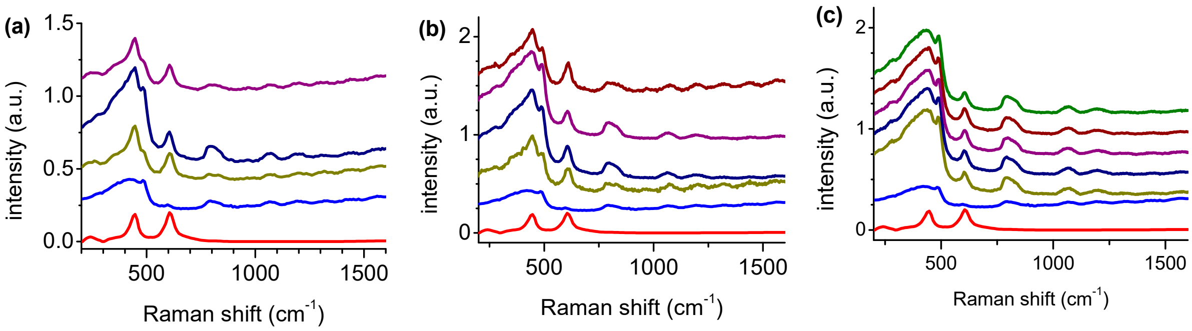

Figure S5. Raw spectra obtained from TiO_2 NPs. (a)Three individual raw Raman spectra acquired with 3 accumulations of 20 s each for the 18 nm TNP; (b) Four raw Raman spectra measured acquired with 10 accumulations of 60 s each for the 12 nm TNP; (c)Five raw Raman spectra measured for measured acquired with 10 accumulations of 60 s each for the 9nm TNP. Blue and red lines are the reference spectra form fused silica substrate and rutile, respectively.
